# The role of a humanities curriculum in a dermatology residency: A qualitative evaluation of a novel “Dermanities” curriculum

**DOI:** 10.1016/j.jdin.2024.01.006

**Published:** 2024-02-21

**Authors:** Melissa Butt, Marisa Riley, Nanjiba Nawaz, Lauren J. Van Scoy, Heather Costigan, Paul Haidet, Alexandra Flamm

**Affiliations:** a Department of Dermatology, Penn State Milton S. Hershey Medical Center, Hershey, Pennsylvania; b Department of Public Health Sciences, Penn State College of Medicine, Hershey, Pennsylvania; c Department of Family and Community Medicine, Penn State College of Medicine, Hershey, Pennsylvania; d College of Medicine, Penn State Health, Hershey, Pennsylvania; e Department of Medicine, Penn State Milton S. Hershey Medical Center, Hershey, Pennsylvania; f Qualitative and Mixed Methods Core, Penn State College of Medicine, Hershey, Pennsylvania; g Department of Humanities, Penn State College of Medicine, Hershey, Pennsylvania; h The Woodward Center for Excellence in Health Sciences Education, Penn State College of Medicine, Hershey, Pennsylvania; i Department of Dermatology, NYU Grossman School of Medicine, New York, New York

**Keywords:** course evaluation, dermatology education, graduate medical education, humanities-based education, qualitative research

*To the Editor:* Humanities -based medical education has demonstrated improved communication, observational skills, and pattern recognition^1^^,^^2^; however, graduate medical education often does not include humanities curricula.^3^ Because of emphasis on visual diagnoses and close patient interactions, dermatology training particularly may benefit from humanities education. Although there have been previous humanities curricula that use fine arts to focus on improving visual diagnostic skills among medical students,^4^ a humanities-inspired graduate medical education curriculum in dermatology has yet to be described in the literature. Consequently, we aimed to implement a novel dermatology-oriented humanities pilot course to determine if these principles would be considered a valuable addition to the currently available curriculum.

The course topics were selected based on relevance to humanities education, previous use in undergraduate and graduate medical education courses, and relevance to dermatology practice.^1^^,^^4^ Full curriculum details are outlined in Table I. The course was delivered to dermatology residents at a single medical center from January to June 2020. Immediately following the course, residents were invited to participate in a 1-hour virtual focus group with a $25.00 stipend for participation in order to evaluate the acceptability of the course. Focus groups were led by a trained qualitative researcher using a semi-structured interview guide, audio-recorded, transcribed, and blinded for analysis.

**Table I:** Outline of “Dermanities” curriculum

Session	Title	Session content	Practice point
1	Perceptions of dermatology in the humanities	• Present depictions of common dermatologic conditions in print, television, and film. • Discuss of perceptions of dermatologic conditions within the lay culture.	Ask one of your patients if he or she has seen their skin condition in a movie/TV show/book/comic, etc. If yes, how did that make them feel? Was it positive or negative?
2	Use of descriptive language: visual thinking strategies (VTS)	• Apply VTS to describe artwork, artwork depicting dermatologic conditions, and dermatologic images.	Practice point: use VTS when evaluating a patient in your clinic: 1. What did you observe? 2. Did it change your practice? 3. Is there anything you noted you may have missed without using VTS?
3	Use of metaphor	• Define metaphor and associated values. • Discuss use of metaphor in poetry. • Assess the use of metaphor in daily communication and in patient-physician communication.	When educating a patient use a metaphor to describe a diagnosis or its treatment. Be prepared to discuss what you used and why you used that metaphor at our next session.
4	Visual-spatial orientation (“Mise en Scene”)	• Define mise en scene and its various elements, utilizing examples within film. • Demonstrate how mise en scene can be utilized within the physician office. • Demonstrate how mise en scene can be utilized within telemedicine.	Find an example of mise en scene being used in clinic. 1. What concept is being used? 2. What is the purpose of its use? 3. Is it being used effectively?
5	Color theory	• Define the different elements of color and color theory. • Appraise how color theory can be utilized within the physician practice space. • Evaluate the role of color theory within dermatology.	Find an example of color theory being used in clinic. 1. What concept is being used? 2. What is the purpose of its use? 3. Is it being used effectively?
6	Cultural competency	• Define culture and cultural competency. • Explore how the definitions of beauty have changed in various historic eras. • Formulate how to incorporate a culturally based systems approach within patient care.	Course conclusion.

A total of 130 comments were analyzed from 7 residents over 2 focus groups. An inductive approach to content analysis was performed using NVivo version 2020.^5^ A codebook was created after reviewing each transcript. Two coders (HC and MB) assigned these codes to the data using the constant comparison method. Intraclass coefficients (Cohen’s kappa) were calculated to identify discrepancies in coding, with a final kappa of 0.72. Coding patterns were reviewed to extract 3 themes and 7 subthemes (Fig 1).

**Fig 1:**
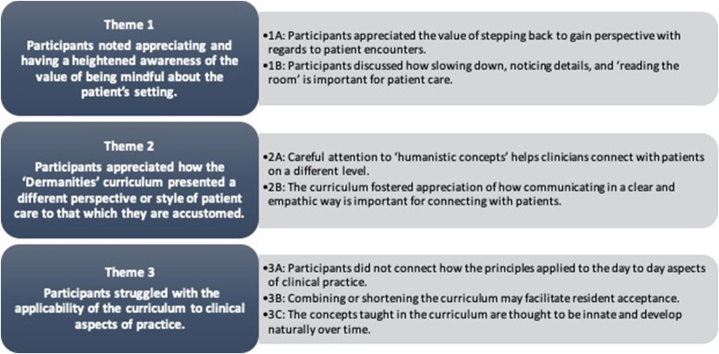
Outline of qualitative themes derived from participant feedback.

Positive course feedback made up the largest portion of comments (*n* = 52, 40.00%), followed by suggestions for improvement (*n* = 50, 38.46%), and negatives or ambivalence about the course (*n* = 24, 18.46%). The most prominent theme was appreciation and heightened awareness regarding patient setting, specifically the value of stepping back to gain perspective regarding patient encounters. The second theme stressed appreciation that the curriculum presented a different perspective to patient care and that careful attention to “humanistic concepts” may help physicians connect with patients on a different level. The third theme showed that participants struggled with the applicability of the curriculum to the daily aspects of clinical practice.

Despite the small sample size, implementing a pilot humanities course within dermatology residency was perceived as beneficial and may enhance residents’ self-awareness, broaden their perspectives about approaches to patient care, and increase interpersonal awareness in patient-physician interactions. However, applications for use of these skills in clinical settings must be emphasized. The development of humanities content can familiarize residents with valuable concepts that are essential to physician training such as competent medical knowledge and empathetic patient care. Results from these pilot data warrant additional exploration on the benefits of implementing humanities-based curricula across different dermatology training programs as well as to evaluate how these principals elicit beneficial changes in clinical understanding and practice.

## Conflicts of interest

None disclosed.
